# Literary Notes and Miscellany

**Published:** 1900-10

**Authors:** 


### LITERARY NOTES AND MISCELLANY.

MESSRS. WILLIAM R. WARNER & COMPANY, of Philadelphia, New York and Chicago, have been advised of the award by the judges of the Paris Exposition, of the highest medal prize, for their justly celebrated pharmaceutical products. This makes the seventeenth World's Fair highest prize, which has been awarded this well known and justly celebrated firm, and we join in congratulations to Messrs. Wm. R. Warner & Co. over their well-merited and unbroken line of victories in competition with the world's manufacturers.

THE Maltine Company, of New York, has presented a new and striking original advertisement, on page xiii. of this issue of the JOURNAL. This "family tree" of maltine suggests a vigorous, healthy growth, which neither the stress of time nor of storms have been able to retard. The house is now twenty-five years old, and its watchword appears to be expansion, for it is growing everyday in strength and size.

THE J. B. Lippincott Company, medical publishers of Philadelphia, have purchased a site and begun the preparation of a new home in place of the old one which was destroyed by fire some months ago. The new location is on East Washington Square, where a fine building will be reared in place of the old-fashioned dwelling houses belonging to the Horace Binney estate. The Lippincott Company is to be congratulated upon the prospects of soon enlarging its long-time established and solid publishing plant.

W. B. SAUNDERS & Co., medical publishers, Philadelphia, desire to announce that they are about to establish a branch of their business in Great Britain. Mr. Saunders has recently spent several weeks in London, where all the arrangements preliminary to the opening of an English house have been completed.

This London branch will be operated in immediate connection with the home establishment, and the same methods that have been so successful in building up the business in this country will be employed in the conduct of this new branch.

Mr. Saunders also announces that he has ready The American Illustrated Medical Dictionary, by W. A. N. Dorland, editor of The American Pocket Medical Dictionary. This is an entirely new and unique work for students and practitioners. It contains more than twice the matter in the ordinary students' dictionary, and yet, by the use of clear, condensed type and thin paper of the finest quality, it forms an extremely handy volume only one and one-half inches thick. It is declared to be a beautiful specimen of the bookmakers' art. Price, $4.50 net; indexed, $5.00.

Saunders & Co. also have ready the following:

*New Books.*—Modern Medicine, by Drs. J. L. Salinger and F. J. Kalteyer, of Jefferson Medical College, Philadelphia. Price, $4.00 net.

Rhinology, Laryngology and Otology, and their Significance in General Medicine, by Dr. E. P. Friedrich, of the University of Leipsig, and Dr. H. Holbrook Curtis, of New York. Price, $2.50 net.

A Text-book of Histology, by Drs. Bohm and Davidoff, of Munich, and Dr. G. Carl Huber, of Ann Arbor, Michigan. Ready in October.

Essentials of Histology, by Dr. Louis Leroy, of Vanderbilt University. Price, $1.00 net.

Surgical Technic for Nurses, by Emily A. M. Stoney, author of "Stoney's Nursing."

*New Editions.*—Anders's Practice of Medicine, 4th edition. Price, $5.50 net.

McFarland's Bacteriology, 3d edition, revised and enlarged. Price, $3.25 net.

Hyde & Montgomery's Venereal Diseases, new enlarged edition. Price, $4.00 net.

American Text-book of Physiology, 2d edition, revised, in two volumes. Volume I. now ready. Price, $3.00 net, per volume.

Saunders's Pocket Formulary, 5th edition, increased in size by over 200 formulæ. Price, $2.00 net.

Garrigues's Diseases of Women, 3d edition. Price, $4.50 net.

DaCosta's Surgery, 3d greatly enlarged edition. Price, $5.00 net.

Stengel's Pathology, 3d edition, revised. Price, $5.00 net.

During the next six months they will issue from four to six volumes in Saunders's Medical Hand-Atlas Series.

---

THE International Journal of Surgery Company, medical publishers of New York, announces the following new books on press. The Treatment of Fractures, by W. L. Estes, A.M., M.D., director and physician and surgeon-in-chief St. Luke's Hospital, South Bethlehem, Pa. This book describes the treatment of fractures in a practical and interesting manner and is replete with original drawings, photographs and skiagraphs. Printed upon heavy book paper and substantially bound in cloth, containing about 250 pages. Price, $2.00.

The Technique of Surgical Gynecology, by Augustin H. Goelet, M.D., professor of gynecology in the New York School of Clinical Medicine, consulting professor of gynecological electro-therapeutics, International Correspondence Schools, Scranton, Pa., etc. This book presents to the reader the technique of gynecological operations in a manner combining simplicity with completeness. The various subjects are individually discussed and graphically illustrated with a large number of drawings and sketches especially prepared. About 300 pages, price, $2.00.

---

THE *Medical Mirror* announced in its September issue that hereafter it will be published simultaneously in New York and St. Louis. On and after September 15th, all communications for the editor should be addressed to Dr. I. N. Love, "The Iroquois," 49 West 44th St., New York City, U. S. A. All business communications, remittances, and exchanges to Love's *Medical Mirror*, St. Louis, U.S.A.

